# Fecal Fungal Dysbiosis in Chinese Patients With Alzheimer’s Disease

**DOI:** 10.3389/fcell.2020.631460

**Published:** 2021-01-28

**Authors:** Zongxin Ling, Manlian Zhu, Xia Liu, Li Shao, Yiwen Cheng, Xiumei Yan, Ruilai Jiang, Shaochang Wu

**Affiliations:** ^1^State Key Laboratory for Diagnosis and Treatment of Infectious Diseases, Collaborative Innovation Center for Diagnosis and Treatment of Infectious Diseases, National Clinical Research Center for Infectious Diseases, The First Affiliated Hospital, School of Medicine, Zhejiang University, Hangzhou, China; ^2^Department of Geriatrics, Lishui Second People’s Hospital, Lishui, China; ^3^Department of Intensive Care Unit, The First Affiliated Hospital, School of Medicine, Zhejiang University, Hangzhou, China; ^4^Institute of Hepatology and Metabolic Diseases, Hangzhou Normal University, Hangzhou, China; ^5^Institute of Translational Medicine, The Affiliated Hospital of Hangzhou Normal University, Hangzhou, China

**Keywords:** Alzheimer’s disease, *Candida*, fungal microbiota, sequencing, TNF-α

## Abstract

Gut bacterial dysbiosis plays a vital role in the development of Alzheimer’s disease (AD). However, our understanding of alterations to the gut fungal microbiota and their correlations with host immunity in AD is still limited. Samples were obtained from 88 Chinese patients with AD, and 65 age- and gender-matched, cognitively normal controls. Using these samples, we investigated the fungal microbiota targeting internal transcribed spacer 2 (ITS2) rRNA genes using MiSeq sequencing, and analyzed their associations with the host immune response. Our data demonstrated unaltered fungal diversity but altered taxonomic composition of the fecal fungal microbiota in the AD patients. The analysis of the fungal microbiota was performed using 6,585,557 high-quality reads (2,932,482 reads from the controls and 3,653,075 from the AD patients), with an average of 43,042 reads per sample. We found that several key differential fungi such as *Candida tropicalis* and *Schizophyllum commune* were enriched in the AD patients, while *Rhodotorula mucilaginosa* decreased significantly. Interestingly, *C. tropicalis* and *S. commune* were positively correlated with IP-10 and TNF-α levels. In contrast, *C. tropicalis* was negatively correlated with IL-8 and IFN-γ levels, and *R. mucilaginosa* was negatively correlated with TNF-α level. PiCRUSt analysis revealed that lipoic acid metabolism, starch and sucrose metabolism were significantly decreased in the AD fungal microbiota. This study is the first to demonstrate fecal fungal dysbiosis in stable AD patients at a deeper level, and to identify the key differential fungi involved in regulating host systemic immunity. The analysis of the fungal microbiota in AD performed here may provide novel insights into the etiopathogenesis of AD and pave the way for improved diagnosis and treatment of AD.

## Introduction

Alzheimer’s disease (AD) is a neurodegenerative disorder characterized by a slow progression, which starts with mild memory loss and culminates in severe impairment of executive and cognitive functions. During the last decades, the prevalence of AD has been rapidly increasing due to the rise in life expectancy worldwide (Scheltens et al., 2016; Gaugler et al., 2019; Alzheimer’s Association, 2020). It is estimated that by 2050, one in every 85 people will be living with AD (Brookmeyer et al., 2007). However, at present there are no mechanistic therapies or disease-modifying therapies available for AD (Honig et al., 2018). As a result, a diagnosis of AD has become one of the most devastating that patients and their families can receive. The financial burden imposed by AD is formidable due to the care needed by the growing number of patients with AD and other dementias. Given the clinical and financial burdens of the disease, AD should be regarded as a global public health priority.

Increasing evidence recognizes AD as a multifactorial and heterogeneous disease with multiple contributors to its pathophysiology, which is not restricted to effects on the central nervous system, but also includes strong interactions with external factors such as the gut microbiota. At autopsy, AD is characterized by amyloid-beta (Aβ) plaques and neurofibrillary tangles (LaFerla et al., 2007; Kinney et al., 2018; Sepulcre et al., 2018; Chen et al., 2020; Wan et al., 2020). Recently, a genome-wide spatial transcriptomic analysis identified an amyloid plaque-induced gene network, suggesting that Aβ plays active roles in the development of AD (Chen et al., 2020). In fact, in the last decades, the progress made by accounts of AD etiopathology focusing on the nervous system remains limited. Recent studies of the gut–brain axis have highlighted the potential roles of the gut microbiota in the development of various brain diseases, including AD. Several studies have found an altered gut microbiota in AD patients, suggesting that the gut microbiota may be involved in AD pathogenesis (Vogt et al., 2017; Zhuang et al., 2018; Liu et al., 2019). Our group has also previously demonstrated that *Clostridium butyricum* and its metabolite butyrate can regulate the expression of Aβ, leading to an amelioration of cognitive deficits and neurodegeneration via modulation of the gut microbiota; these can therefore be considered to be potential psychobiotics (Sun et al., 2019a,b; Sun J. et al., 2020). Furthermore, a phase 3 clinical trial conducted in China by another group found that administering oligomannate led to a solid and consistent improvement in cognition in AD patients, suppressing gut dysbiosis and the associated phenylalanine/isoleucine accumulation, harnessing neuroinflammation, and reversing cognitive impairment (Wang et al., 2019). These findings indicate that gut dysbiosis can promote neuroinflammation during the progression of AD, while restoration of the gut microbiota may be a novel strategy for treating AD.

The gut microbiota is composed of a variety of microorganisms, including bacteria, viruses, fungi, and archaea. However, previous studies have mainly focused on the bacterial diversity and composition of the gut microbiota, while the fungal microbiota has not been explored extensively. Fungi are suggested to influence intestinal health and disease by suppressing the outgrowth of potential pathobionts, promoting immunoregulatory pathways, and modulating host metabolism (Huseyin et al., 2017; Ni et al., 2017; Sam et al., 2017; Chin et al., 2020). Several clinical studies have identified a distinct fungal microbiota dysbiosis in inflammatory bowel disease (IBD), primary sclerosing cholangitis, asthma, type 2 diabetes mellitus, chronic liver diseases, Parkinson’s disease and other neurological diseases, and even colorectal cancer (Hoarau et al., 2016; Sokol et al., 2017; Forbes et al., 2018; Coker et al., 2019; Cirstea et al., 2020; Jayasudha et al., 2020; Jiang et al., 2020; Lemoinne et al., 2020; Qiu et al., 2020; van Tilburg Bernardes et al., 2020; Ventin-Holmberg et al., 2020). Studies of animal models have found that commensal fungi can activate host-protective immune pathways related to epithelial barrier integrity, but can also induce reactions that contribute to events associated with IBD (Iliev and Cadwell, 2020). In addition, by interacting with the bacteriome and/or virome, the gut fungal communities appears to be a cofactor in inflammation and in the host immune response, and therefore may contribute to various disease progression. Therefore, alterations to the fungal microbiota might actively contribute to the development of AD. In this study, we employed fungal-specific internal transcribed spacer (ITS) amplicon sequencing of a cross-sectional AD cohort to investigate associations between the fungal gut microbiota and AD using the 16S rRNA high-throughput gene MiSeq platform. Furthermore, we performed correlation analysis between fungal taxa and clinical indicators to decipher their possible roles in the pathogenesis of AD.

## Materials and Methods

### Subject Enrollment

A total of 88 Chinese patients with well-controlled AD, whose diagnoses were based on the criteria of the National Institute of Neurological and Communicative Diseases and Stroke/AD and Related Disorders Association, were recruited from Lishui, Zhejiang province (China) from February 2019 to November 2019, along with 65 cognitively normal subjects as controls. The cognitive and functional status of each subject was scored using the Mini-Mental State Examination (MMSE, Chinese version), the current version in the Wechsler Adult Intelligence Scale series (WAIS-IV, published in 2008), and the Barthel Index of instrumental activities of daily living. Each participant was scanned using magnetic resonance imaging (MRI), with all AD patients diagnosed as showing brain atrophy. The detailed demographic data and medical history (including hypertension, diabetes mellitus, hypercholesterolemia, coronary heart disease, diarrhea, and constipation) were collected using a set of questionnaires. Exclusion criteria included: family history of dementia; any kind of other neurodegenerative disease such as Parkinson’s disease; confirmed mental illness such as schizophrenia; any kind of tumor; antibiotic, prebiotic, probiotic, or synbiotic administration during the previous month; known active infections such as viral, bacterial, or fungal infections; and other diseases such as inflammatory bowel disease, irritable bowel syndrome, or other autoimmune diseases. The protocols for the study were approved by the Ethics Committee of Lishui Second People’s Hospital (Zhejiang, China) and written informed consent was obtained from each of the subjects or their guardian before enrollment.

### Fecal Sample Collection and DNA Extraction

Approximately 2 g of fresh fecal sample was collected from each subject in a sterile plastic cup, and stored at −80°C after preparation within 15 min, until its subsequent use. Metagenomic DNA was extracted from 300 mg homogenized feces using a QIAamp DNA Stool Mini Kit (QIAGEN, Hilden, Germany) according to the manufacturer’s instructions, with additional glass-bead beating steps performed using a Mini-Beadbeater (FastPrep; Thermo Electron, Boston, MA, United States). The amount of DNA was determined using a NanoDrop ND-1000 spectrophotometer (Thermo Electron). The integrity and size were verified by electrophoresis on a 1.0% agarose gel containing 0.5 mg/ml ethidium bromide. All DNA samples were stored at −20°C prior to further analysis.

### Amplicon Library Construction and Sequencing

Amplicon libraries were constructed using Illumina sequencing-compatible and barcode-indexed fungal PCR primers ITS3 (5′-GATGAAGAACGYAGYRAA-3′) and ITS4 (5′-TCCTCCGC TTATTGATATGC-3′), which target ITS2 rRNA genes (Degnan and Ochman, 2012). All PCR reactions were performed using HiFi HotStart ReadyMix (KAPA Biosystems) according to the manufacturer’s protocol and approximately 50 ng extracted DNA per reaction. Thermocycling conditions were set at 95°C for 1 min, 53°C for 1 min, then 72°C for 1 min for 30 cycles, followed by a final extension at 72°C for 5 min. All PCR reactions were performed in 50 μl triplicates and combined after PCR. The amplicon library was prepared using a TruSeq DNA sample preparation kit (Illumina, San Diego, CA, United States). Prior to sequencing, the PCR products were extracted with the MinElute Gel Extraction Kit (QIAGEN) and quantified on a NanoDrop ND-1000 spectrophotometer (Thermo Electron) and a Qubit 2.0 Fluorometer (Invitrogen). The purified amplicons were then pooled in equimolar concentrations and the final concentration of the library was determined using the Qubit 2.0 Fluorometer. Negative DNA extraction samples (lysis buffer and kit reagents only) were amplified and sequenced as contamination controls. Sequencing was performed on a MiSeq instrument (Illumina) using a 300 × 2 V3 kit together with the PhiX Control V3 library (Illumina) (Ling et al., 2019; Wang et al., 2019). MiSeq sequencing and library construction were performed by technical staff at Hangzhou KaiTai Bio-lab.

### Bioinformatic Analysis

The ITS sequence dataset generated by the MiSeq run were first merged and demultiplexed into per-sample data using QIIME version 1.9.0 with default parameters (Caporaso et al., 2010). Chimera sequences were detected and removed using USEARCH version 7 software based on the UCHIME algorithm (Edgar et al., 2011). The open-reference operational taxonomic unit (OTU) pick was then performed using USEARCH version 7 referenced against the Greengenes database version 13.8 at 97% sequence similarity (Edgar, 2010; McDonald et al., 2012). OTUs containing a number of sequences <0.005% of the total number of sequences were discarded, as recommended (Navas-Molina et al., 2013). This resulted in an OTU table, which was used for subsequent downstream analysis.

To perform the taxonomic assignment, the most abundant sequence from each OTU was chosen as the representative sequences from that OTU. Taxonomic assignment of individual datasets was performed by classifying the data according to the UNITE database^1^ (Abarenkov et al., 2010). Alpha diversity was calculated based on the sequence similarity at the 97% level using QIIME software and Python scripts to calculate a range of estimators, including: index of observed species, abundance-based coverage estimator (ACE), Chao1 estimator, Shannon, Simpson, evenness, and PD whole tree. Sequence coverage was assessed in Mothur software based on calculating rarefaction curves and Good’s coverage (Good, 1953; Schloss et al., 2009). Beta diversity was estimated based on the Jaccard, Bray–Curtis, unweighted UniFrac, and weighted UniFrac distances calculated with 10× subsampling in QIIME. These distances were visualized following principal coordinate analysis (PCoA) of the data (Lozupone and Knight, 2005). Hierarchical clustering was performed and a heatmap was generated using a customized script developed in the R statistical package, with Spearman’s rank correlation coefficient as the distance measure. The output file was further analyzed using the Statistical Analysis of Metagenomic Profiles (STAMP) software package version 2.1.3 (Parks et al., 2014).

To perform the predictive functional analyses, PiCRUSt software version 1.0.0 was used to identify predicted gene families and associated pathways from the inferred metagenomes of taxa of interest identified during the compositional analyses; this analysis is based on the close link between phylogeny and function (Langille et al., 2013). Predicted functional genes were categorized based on the Clusters of Orthologous Groups (COG) database and on the Kyoto Encyclopedia of Genes and Genome (KEGG) orthology (KO), and then compared across patient groups using STAMP. Pathways and enzymes were assigned using the KEGG database options built into the pipeline. Pathways that were non-prokaryotic, had <2 sequences in each cohort, or had a difference in mean proportions <0.1% were excluded from the analysis. The characterization of microorganismal features differentiating the gastric microbiota was performed using the linear discriminant analysis (LDA) effect size (LEfSe) method^2^ for biomarker discovery, which emphasizes both statistical significance and biological relevance (Segata et al., 2011). Based on a normalized relative abundance matrix, the LEfSe method uses the Kruskal–Wallis rank sum test to detect features with significantly different abundances between assigned taxa and then performs LDA to estimate the effect size of each feature. A significant alpha threshold of 0.05 and an effect size threshold of 3 were used to identify all of the biomarkers discussed in this study.

### Systemic Inflammatory Cytokines Analysis

Serum samples were obtained from the participants using their fasting blood in the early morning. The following cytokines were quantified using a 27-plex magnetic bead based immunoassay kit (Bio-Rad, Hercules, CA, United States): interleukin-1β (IL-1β), IL-1 receptor antagonist (IL-1ra), IL-2, IL-4, IL-5, IL-6, IL-7, IL-8, IL-9, IL-10, IL-12 (p70), IL-13, IL-15, IL-17, eotaxin, fibroblast growth factor-basic (FGF-basic), granulocyte colony-stimulating factor (G-CSF), granulocyte-macrophage colony-stimulating factor (GM-CSF), interferon gamma (IFN-γ), interferon gamma-inducible protein 10 (IP-10), monocyte chemotactic protein-1 (MCP-1), macrophage inflammatory protein-1α (MIP-1α), platelet-derived growth factor (PDGF-bb), MIP-1β, regulated upon activation normal T-cell expressed and secreted (RANTES), tumor necrosis factor-alpha (TNF-α), and vascular endothelial growth factor (VEGF). The Bio-Plex 200 system (Bio-Rad) was used to analyze Bio-Rad 27-plex human group I cytokines, with the Bio-Plex assay performed according to the manufacturer’s directions. The results were expressed as picograms per milliliter (pg/mL) using standard curves integrated into the assay and the Bio-Plex Manager v5.0 software (Bio-Rad), yielding reproducible intra-and inter-assay CV values of 5–8%.

### Statistical Analysis

White’s nonparametric *t*-test, the independent *t*-test, or the Mann-Whitney *U*-test were applied to analyze continuous variables. Pearson’s chi-square test or Fisher’s exact test were used to analyze categorical variables between groups. Spearman’s rank correlation test was used to perform correlation analyses. Statistical analysis was performed using SPSS version 19.0 (SPSS Inc., Chicago, IL, United States) and STAMP version 2.1.3 (Parks et al., 2014). R and GraphPad Prism v6.0 software were used to prepare graphs. All of the tests of significance performed were two sided, with *p* < 0.05 or corrected *p* < 0.05 considered statistically significant.

### Accession Number

The sequence data from this study are deposited in the GenBank Sequence Read Archive with the accession number SRP292858.

## Results

### Subject Characteristics

Eighty-eight stable AD patients and 65 age- and gender-matched cognitively normal, healthy controls were enrolled in the present study (Table 1). All participants were older than 65 years of age. There were no significant differences between the healthy controls and the AD patients in terms of gender, body mass index, drinking, or smoking, or in terms of comorbidities with hypertension, hypercholesterolemia, diabetes mellitus, or coronary heart disease (all *p*s > 0.05). However, MMSE, WAIS, and Barthel scores were clearly lower in AD patients than in the healthy controls (all *p*s < 0.05).

**TABLE 1:** The fundamental information of subjects.

Parameters	AD patients (*n* = 88)	Healthy control (*n* = 65)
Age (y)	74.28 ± 8.89	73.58 ± 8.15
Gender (male/female)	40/48	32/33
BMI (Mean ± SD)	23.20 ± 3.25	23.68 ± 3.48
Antibiotics use, no.	0	0
Complications, no.		
Hypertension	30	19
Diabetes mellitus	13	8
Hypercholesterolemia	12	8
Coronary heart disease	11	6
Diarrhea	2	3
Constipation	6	4
Cognitive and functional status		
MMSE Score*	4.35 ± 5.89	26.85 ± 3.75
WAIS Score*	36.25 ± 16.84	91.25 ± 11.28
Barthel Score*	22.38 ± 24.20	77.80 ± 9.85

BMI, body mass index; SD, standard deviation; no., numbers; MMSE, Mini-Mental State Examination; WAIS, Wechsler Adult Intelligence Scale.

* *p* < 0.05.

### Unaltered Overall Structure of the Fungal Microbiota in Stable AD

**FIGURE 1:**
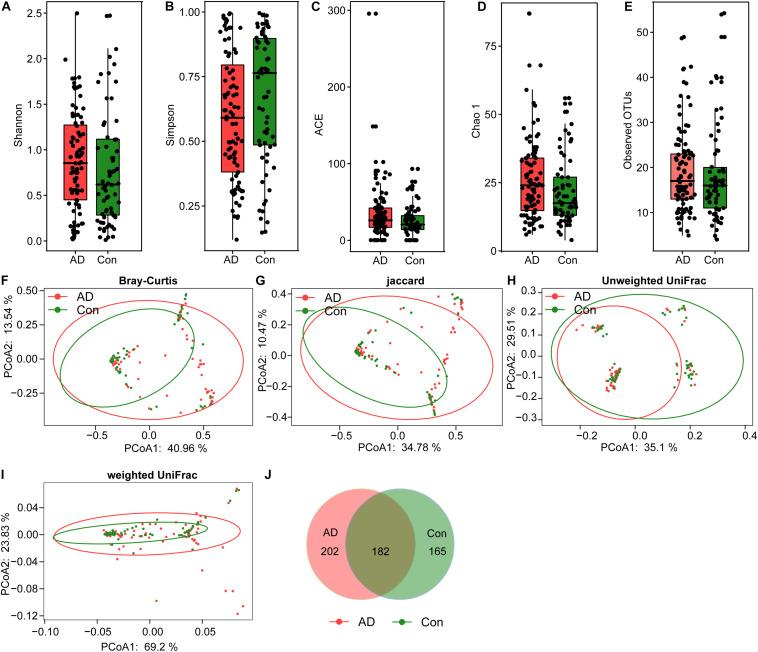
Fungal diversity and richness of the fecal microbiota in Chinese AD patients. The diversity indices of Shannon **(A)** and Simpson **(B)**, and the richness indices of the observed species **(C)**, ACE **(D)**, and Chao1 **(E)** were used to evaluate the overall structure of the fungal microbiota in the stable AD patients and the healthy controls. The data are presented as mean ± standard deviation. Unpaired *t*-tests (two tailed) were used to analyze the variation between the groups. Principal coordinate analysis (PCoA) plots of individual fungal microbiota based on Bray–Curtis **(F)**, Jaccard **(G)**, and unweighted **(H)** and weighted **(I)** UniFrac distances in the AD patients and the healthy controls. Each symbol represents a sample. The Venn diagram illustrates the overlap of OTUs in the fungal microbiota between the two groups **(J)**.

In the present study, 6,585,557 high-quality reads (2,932,482 reads from the controls and 3,653,075 from the AD patients) were obtained for subsequent analysis of the fungal microbiota, with an average of 43,042 reads per sample. Good’s coverage was 99.99% in healthy controls and 99.98% in AD patients, respectively, suggesting that most of the fungal phylotypes (546 OTUs) in the AD-associated fungal microbiota were successfully identified. Interestingly, the calculated fungal alpha-diversity indices, including the Shannon and Simpson indices, show no significant changes of the AD-associated fungal microbiota relative to that of the healthy controls (Figures 1A,B); however, there was a trend toward increasing fungal diversity in the stable AD patients. In terms of the richness indices, there were no significant changes in ACE, Chao1, and observed OTUs in stable AD patients compared with healthy controls (Figures 1C–E; all *p*s > 0.05). Rarefaction plots reached a plateau for fungal species in both the AD and the control samples. We next assessed and compared the beta diversity of the fungal microbiota in AD patients to that in healthy controls based on the Bray–Curtis, Jaccard, unweighted UniFrac, and weighted UniFrac algorithms. The AD and control groups could not be divided into different clusters (Adonis test, *p* > 0.01; Figures 1F–I). In addition, the Venn diagram showed more unique phylotypes in AD patients than those in healthy controls (Figure 1J). Taken together, the alpha- and beta-diversity analyses demonstrate that the overall structure of the fungal microbiota in stable AD patients did not change obviously compared with that in the healthy controls.

### Taxonomic Alterations of Fecal Fungi in Stable AD

**FIGURE 2:**
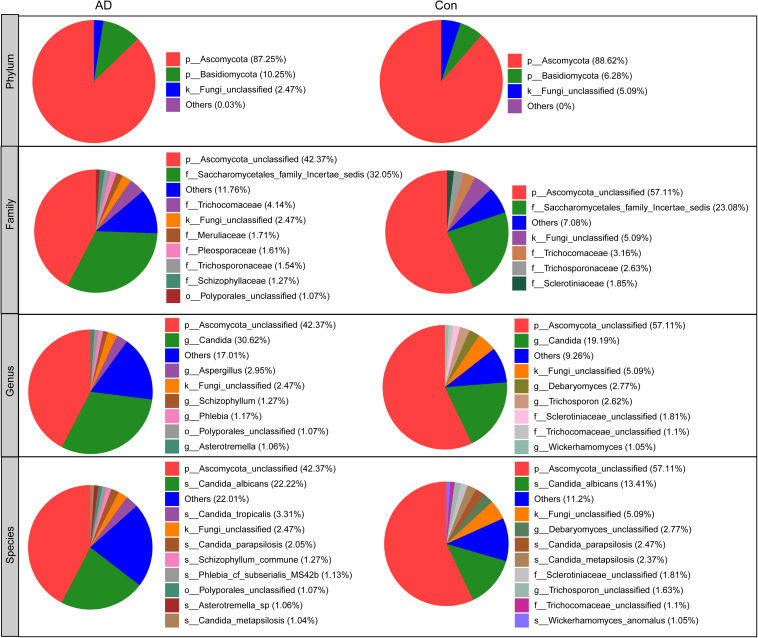
Overall fungal microbiota composition in stable AD patients and healthy controls in phylum, family, genus, and species levels.

**FIGURE 3:**
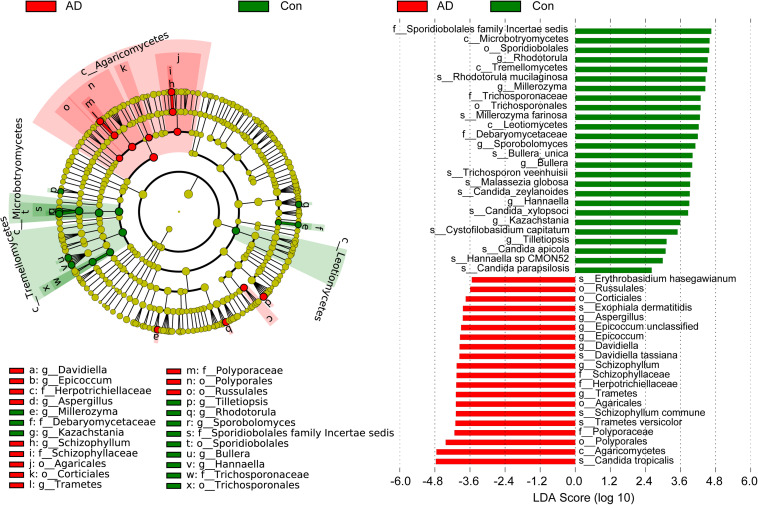
Differential fungal taxa between the stable AD patients and the healthy controls. The LEfSe identified the taxa with the greatest differences in abundance between the AD patients and healthy controls. Only the taxa meeting a significant LDA threshold value of >2 are shown. These differential fungi could be used as potential biomarkers to distinguish AD patients from healthy controls.

The compositions of the fungal microbiota in the stable AD patients and healthy controls were assessed at different taxonomic levels (Figure 2). Overall, four phyla, 20 classes, 52 orders, 97 families, 148 genera, and 247 species were identified by this sequencing analysis. Among the fungal taxa, the phylum Ascomycota dominated the fungal microbiota, while Basidiomycota was observed as the second most abundant phylum in both the AD and control groups. At the family level, Saccharomycetales family Incertae sedis, Trichocomaceae, Meruliaceae, Pleosporaceae, Trichosporonaceae, Schizophyllaceae, and Sclerotiniaceae were found to be dominant in the fungal microbiota, when unclassified fungal taxa were excluded. Interestingly, we found that a greater number of fungal families were observed in the AD patients compared with the healthy controls. At the genus level, classified genera including *Candida*, *Aspergillus*, *Debaryomyces*, *Trichosporon*, *Wickerhamomyces*, *Schizophyllum*, *Phlebia*, and *Asterotremella* were abundant in the fungal microbiota, both in the AD patients and the healthy controls. At the species level, fungal taxa including *Candida albicans*, *Candida tropicalis*, *Candida parapsilosis*, *Schizophyllum commune*, *Phlebia cf. subserialis* MS42b, *Asterotremella* sp., *Candida metapsilosis*, and *Wickerhamomyces anomalus* were observed and classified. *C. albicans*, *C. tropicalis*, and *C. parapsilosis* were the most abundant species in the fungal microbiota, both in the AD patients and the healthy controls. Using the LEfSe method, discriminant analysis showed that many key taxa were clearly different between the AD and control groups (LDA score >2, *p* < 0.05; Figure 3). Although the Basidiomycota/Ascomycota ratio is considered to be an indicator of fungal dysbiosis, we found no significant differences in these two abundant phyla between the AD patients and the healthy controls (Coker et al., 2019). The LEfSe analysis revealed that most of the differential fungi could be classified at the species level. *C. parapsilosis*, *Hannaella* sp. CMON52, *C. apicola*, *Cystofilobasidium capitatum*, *C. xylopsoci*, *C. zeylanoides*, *Malassezia globosa*, *Trichosporon veenhuisii*, *Bullera unica*, *Millerozyma farinosa*, and *Rhodotorula mucilaginosa* were enriched in the healthy controls, while *C. tropicalis*, *Trametes versicolor*, *S. commune*, *Davidiella tassiana*, *Exophiala dermatitidis*, and *Erythrobasidium hasegawianum* were prevalent in stable AD patients. The abundances of the most numerous *Candida* species, such as *C. albicans*, did not show obvious changes between the two groups. Despite most of these differential species not being abundant, our results do nevertheless indicate fungal dysbiosis in stable AD patients.

### Fungal Functional Alterations in AD

**FIGURE 4:**
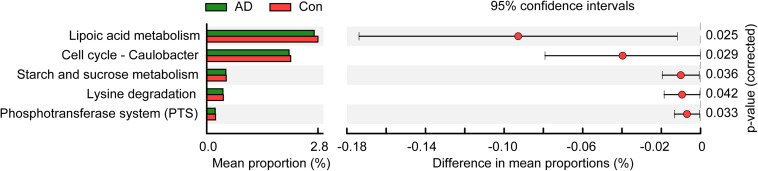
PiCRUSt-based examination of the fungal microbiome of the stable AD patients and the healthy controls. The different fungal functions were evaluated between them based on two-sided Welch’s *t*-test. Comparisons between the groups for each KEGG functional category (level 3) are shown by percentage. The Benjamini–Hochberg method was used for multiple testing correction based on the false discovery rate (FDR) through STAMP.

To identify metabolic and functional changes in the fungal microbiota between the AD patients and the controls, we used PiCRUSt to analyze the functional potential of the microbiota based on closed-reference OTU picking. We compared 105 KEGG pathways at level 3 and identified five KEGG categories with clearly differential abundances between the AD patients and the controls. The following KEGG categories decreased prominently in stable AD patients (*p* < 0.05; Figure 4): “lipoic acid metabolism,” “cell cycle – Caulobacter,” “starch and sucrose metabolism,” “lysine degradation,” and “phosphotransferase system (PTS).” These fungal functional alterations might participate in the pathogenesis and development of AD.

### Correlations Between Key Differential Fungi and Host Immunity

**FIGURE 5:**
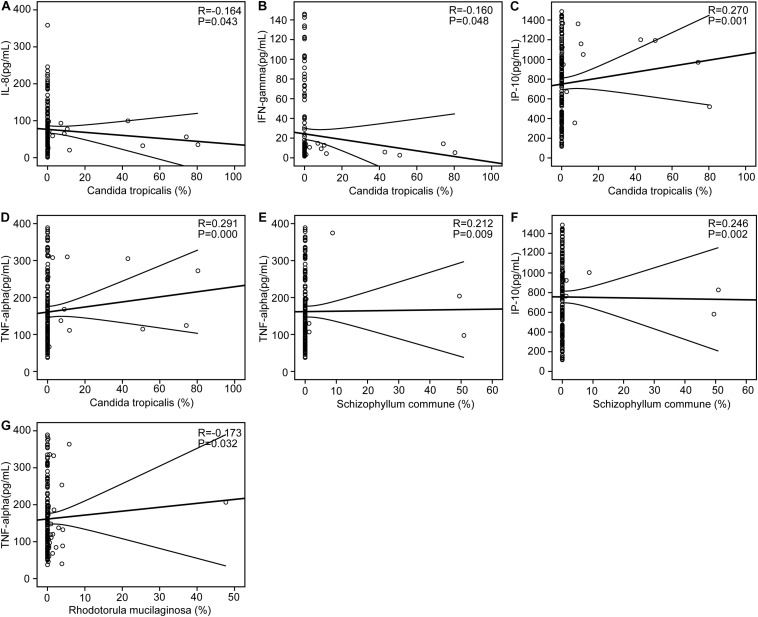
Correlations between pro- and anti-inflammatory cytokines and chemokines with altered concentrations and the relative abundance of the key differential fungi. Correlation between the relative abundance of *Candida tropicalis* and the levels of IL-8 **(A)**, IFN-γ **(B)**, IP-10 **(C)**, and TNF-α **(D)**; the relative abundance of *Schizophyllum commune* and the levels of TNF-α **(E)** and IP-10 **(F)**; the relative abundance of *Rhodotorula mucilaginosa* and the levels of TNF-α **(G)**. Spearman’s rank correlation (R) and probability (P) were determined to evaluate the statistical importance.

We found that the AD-associated clinical indicators we examined, including the MMSE, WAIS, and Barthel scores, were significantly lower in the stable AD patients compared with the healthy controls (*p* < 0.01). Using the Bio-Plex Pro human cytokine group I panel 27-plex analysis, we found that, in the AD patients relative to the healthy controls, levels of anti-inflammatory cytokines (such as IFN-γ) and several chemokines (such as IL-8, MCP-1, and MIP-1a) were significantly lower (*p*s < 0.05), while levels of pro-inflammatory cytokines (such as TNF-α) were markedly higher (*p*s < 0.05); furthermore, the level of IP-10 was also lower in the AD patients (*p*s < 0.05). Next, we investigated correlations between the key differential fungi and the altered cytokines using Spearman’s rank correlation (Figure 5). We found that the enriched abundance of *C. tropicalis* in AD correlated negatively with levels of IL-8 and IFN-γ, but correlated positively with levels of IP-10 and TNF-α (*p*s < 0.05). However, the abundance of *C. parapsilosis*, which was prevalent in healthy controls, was not correlated with altered cytokine levels. The abundance of another AD-enriched fungi, *S. commune*, was positively correlated with levels of TNF-α and IP-10 (*p*s < 0.05). The abundance of *R. mucilaginosa*, a non-abundant fungi that was enriched in healthy controls, was negatively correlated with the level of TNF-α (*p* < 0.05). Thus, these results indicate that the altered key differential fungi regulated the systemic immune response in the AD patients, and may actively contribute to the development and progression of AD.

## Discussion

In the present study, we observed fungal microbiota dysbiosis in Chinese patients with stable AD for the first time. Using high-throughput sequencing techniques, we found that alterations to several key differential fungi were associated with AD, and showed clear correlations with the host immune response. These altered fungal taxa may play vital roles in the development and progression of AD.

The human gut microbiota is a complex and diverse ecosystem composed of bacteria, fungi, viruses, and archaea (Hamad et al., 2017). Of course, bacteria represent the majority of the microbial communities that inhabit the human gut, and their roles and mechanisms in human health and disease have been elucidated extensively. Maintaining a healthy balance of gut bacteria can promote good health, and indeed several previous studies have reported that AD pathology is closely correlated with alterations in gut bacterial profiles (Vogt et al., 2017; Zhuang et al., 2018; Li B.Y. et al., 2019; Liu et al., 2019). This suggests that human gut bacteria may play crucial roles in the etiopathology of AD, such that these key differential bacteria could be used as potential targets for non-invasive diagnosis and treatment of AD. Unlike the bacterial community in the human gut, the composition and diversity of the fungal microbiota remains largely unexplored, because of the relatively low abundance of fungi in the human gut, combined with their neglect in research employing culture-based and molecular analyses (Marchesi, 2010). Nevertheless, intestinal fungi represent an important component of the microbiota in the human gastrointestinal tract that interacts with gut immune cells to maintain a healthy gut (Leonardi et al., 2018; Bacher et al., 2019). The gut fungal microbiota has recently been recognized as a novel and important player in the pathophysiology of intestinal and extraintestinal diseases (Huseyin et al., 2017), and is known to have a profound influence in modulating local as well as peripheral immune responses (Li X.V. et al., 2019). Because of the relatively low abundance of fungi in the human gastrointestinal tract (comprising only 2% of the human gut microbiota) (Anandakumar et al., 2019), little is known about their ability to influence human health and disease. With the advent of deep sequencing technologies, the composition and diversity of the fungal microbiota has been revealed, deepening and clarifying our understanding of the roles and mechanisms of intestinal fungi in host homeostasis (Iliev and Leonardi, 2017; Scheffold et al., 2020; Zhang et al., 2020; Zou et al., 2020). Recent studies have unveiled the potential roles that fungi play in modulating host immune homeostasis and inflammatory disease (Standaert-Vitse et al., 2006; Wheeler et al., 2016; Sokol et al., 2017; Yang et al., 2017; Cirstea et al., 2020; Huang et al., 2020; Jiang et al., 2020). However, the fungal microbiota is still a novel and emerging topic of research that continues to lag behind the level of research and understanding we have of the bacteriome. Prior to this study, there have not been any studies focusing on the roles and mechanisms of gut fungal communities in AD, nor in any other neurodegenerative disorder. Nevertheless, the in-depth analysis of the AD-associated fungal microbiota present here might provide novel insights into the development, progression, and treatment of AD.

Fungal species detected in the human body mainly belong to three different phyla: Ascomycota, Basidiomycota, and Zygomycota (Gouba et al., 2013). Most fungal species can be considered commensal or mutualistic, while several yeast and filamentous fungi have been proved to be pathogenic (Parfrey et al., 2011). In the present study, we obtained more than 40,000 reads per sample, allowing us to characterize fungal diversity and composition in depth. In terms of the overall structure of the AD-associated fungal microbiota, we found no significant differences in alpha- and beta-diversity in the stable AD patients compared with the healthy controls. The finding of a lack of alteration to the fungal diversity differs from the alterations to bacterial diversity observed in AD patients; however, the results of the fungal microbiota analysis presented here are consistent with those observed in Parkinson’s disease (Cirstea et al., 2020). Other studies of the fungal microbiota in healthy adults have reported similar findings, including that the fungal microbiota is dominated by yeast and exhibits low fungal diversity and abundance, and high inter-individual variability (Nash et al., 2017; Auchtung et al., 2018). Liu et al. (2019) found that fecal microbial diversity decreased in AD patients compared with healthy controls. Given the significant inter-subject variability in our data, PCoA based on the Bray–Curtis, Jaccard, unweighted UniFrac, and weighted UniFrac algorithms was unable to divide the two groups into different clusters. In contrast, a prior study reported significant compositional differences in the intestinal bacteriome between AD patients and healthy controls based on PCoA using the Bray–Curtis dissimilarity (Liu et al., 2019). Thus, the unaltered diversity of the AD-associated fungal microbiota observed in this study might imply that stable AD does not change the overall structure of the fungal microbiota.

In contrast to bacterial 16S analyses, for which well-established, commonly accepted databases of sequences are available, fungal ITS analysis is relatively undeveloped (Tang et al., 2015), with the UNITE database (see text footnote 1) probably being the most commonly used fungal ITS database. Overall, we were able to classify our fungal ITS reads into different taxonomic levels, but for many of these reads, it was not possible to assign them into specific taxa based on the UNITE database. In the present study, we identified three phyla in the AD-associated fungal microbiota, including Ascomycota, Basidiomycota, and Zygomycota, with most of sequences being assigned to the phylum Ascomycota. Recently, Coker et al. demonstrated that the Ascomycota/Basidiomycota ratio can be considered to be an indicator of fungal dysbiosis (Coker et al., 2019). However, in the present study, we found no significant difference in the Ascomycota/Basidiomycota ratio between the stable AD patients and the healthy controls. This may relate to the stable AD status of the patients, who were not receiving drug treatment. At the genus level, eight genera, namely *Candida*, *Aspergillus*, *Debaryomyces*, *Trichosporon*, *Wickerhamomyces*, *Schizophyllum*, *Phlebia*, and *Asterotremella* were the most abundant in the fecal fungal microbiota and exhibited different levels of prevalence in the AD patients compared with the healthy controls. Specifically, we found that *Aspergillus*, *Schizophyllum*, and *Epicoccum* were enriched in the AD patients, while *Rhodotorula*, *Millerozyma*, *Sporobolomyces*, *Bullera*, *Hannaella*, *Kazachstania*, and *Tilletiopsis* were more prevalent in the healthy controls. These alterations to fungal microbiota composition suggest fungal dysbiosis in the AD-associated fungal microbiota. Furthermore, LEfSe analysis identified several AD-enriched fungal species, including *C. tropicalis*, *Trametes versicolor*, *S. commune*, *Davidiella tassiana*, *Exophiala dermatitidis*, and *Erythrobasidium hasegawianum*, which belong to the genera mentioned above. In contrast, other species, such as *C. parapsilosis*, *Hannaella* sp. CMON52, *C. apicola*, and *R. mucilaginosa*, were prevalent in the healthy controls. Taken together, these species could be used as potential biomarkers for the non-invasive diagnosis of AD. As with bacteria, fungi can be beneficial to host immunity, but they can also exert deleterious effects under pathological conditions associated with disease. Among the altered fungal species identified in the present study, *C. tropicalis*, which is one of the most abundant pathogenic species in the central nervous system (Sanches et al., 2019), was found to be increased significantly in AD patients. Furthermore, we found that the abundance of *C. tropicalis* was negatively correlated with levels of IL-8 and IFN-γ, and positively correlated with those of IP-10 and TNF-α. Our data suggest that *C. tropicalis* might participate in actively regulating the host systemic immune response. Similarly, *S. commune*, a sap-rot Basidiomycota and cosmopolitan species, also exhibited immunomodulatory properties. As with *C. tropicalis*, we found that the enriched abundance of *S. commune* in AD patients correlated positively with levels of IP-10 and TNF-α. In contrast, the abundance of another species, *R. mucilaginosa*, was reduced significantly in the AD-associated fungal microbiota. Our correlation analysis found that the abundance of *R. mucilaginosa* correlated negatively with the level of TNF-α. In addition, the inferred function of the fungal microbiota also changed significantly in the AD patients. Five KEGG pathways were significantly decreased in AD patients, namely: “lipoic acid metabolism,” “cell cycle – Caulobacter,” “starch and sucrose metabolism,” “lysine degradation,” and “phosphotransferase system (PTS).” Previous studies have demonstrated that lipoic acid can function as a novel anti-inflammatory and neuroprotective treatment for AD and related dementias (Holmquist et al., 2007; Maczurek et al., 2008; Sancheti et al., 2014). Sun C. et al. (2020) found that the biological pathway “starch and sucrose metabolism” was associated with serum metabolomic biomarkers that were able to distinguish AD patients from healthy controls. Of course, the relationships between alterations to key functional fungi (especially non-abundant fungi) or inferred functions and AD are still unclear. Collectively, alterations to the composition of the fungal gut microbiota, especially to key functional fungi, and changes in inferred functions actively participate in the development and progression of AD by regulating the host immune response and modulating host metabolic processes.

This study of the fungal microbiota in AD is the first to be conducted, but it did have several limitations. Firstly, the fungal ITS sequencing-based community analysis targeted ITS3/ITS4 with specific PCR primers, which successfully enabled ITS sequencing reads with an average length of nearly 350 bp. However, many ITS sequences could not be correctly annotated and were instead simply annotated as “fungi,” which affected the subsequent analyses of these data. Longer ITS sequences, metagenomic sequences, or the use of well-established fungal databases might help to improve taxonomic assignment. Secondly, most of the fungi identified belonged to non-abundant fungal taxa (with low relative abundance) and could not be detected in all samples, exhibiting a low detection rate. The significant inter-subject variations might have influenced the identification of clinically important fungal species. Thirdly, contamination from food could not be completely excluded in the process of collecting feces; thus, several of the fungal species identified might be associated with the food supplement. Fourthly, AD patients with a newly diagnosed onset were not enrolled in our study. Including these patients in the study might have allowed changes in the abundance patterns of the fungal microbiota to be understood more clearly.

In summary, the present study was the first to analyze the fungal microbiota in AD patients. Although fecal fungal diversity did not change significantly between the AD patients and the healthy controls, the composition of the fungal microbiota was significantly altered. Several key fungal species, including *C. tropicalis*, *Trametes versicolor*, *S. commune*, *Davidiella tassiana*, *Exophiala dermatitidis*, *Erythrobasidium hasegawianum*, were enriched in the AD-associated fungal microbiota, while abundances of *C. parapsilosis*, *Hannaella* sp. CMON52, *C. apicola*, and *R. mucilaginosa* clearly decreased. Key functional fungi, such as *C. tropicalis*, *S. commune*, and *R. mucilaginosa*, were shown to actively participate in regulating the host systemic immune response. The large, case–control study presented here provides novel insights in the etiopathogenesis of AD and paves the way for improved diagnosis and treatment of AD in the future.

## Data Availability Statement

The original contributions presented in the study are publicly available. This data can be found here: GenBank Sequence Read Archive with the accession number SRP292858.

## Ethics Statement

The studies involving human participants were reviewed and approved by the Ethics Committee of Lishui Second People’s Hospital (Zhejiang, China). The patients/participants provided their written informed consent to participate in this study.

## Author Contributions

ZL, SW, and MZ conceived and designed the experiments. ZL, XY, YC, LS, XL, RJ, and MZ performed the experiments. ZL, XY, LS, and MZ analyzed the data. ZL, XL, and LS wrote and edited the manuscript. All authors read and approved the final manuscript.

## Conflict of Interest

The authors declare that the research was conducted in the absence of any commercial or financial relationships that could be construed as a potential conflict of interest.

## Footnotes

1 http://unite.ut.ee/repository.php

2 http://huttenhower.sph.harvard.edu/galaxy/
